# Effects of *Qihuang* Needling on Motor Function for Patients With Parkinson's Disease: Study Protocol for a Multicenter, Randomized Controlled Trial

**DOI:** 10.3389/fneur.2022.902170

**Published:** 2022-06-02

**Authors:** Lian-Sheng Yang, Yang-Mei Li, Dan-Feng Zhou, Bai-Ming Zhao, Shu-Zhen Zheng, Zhen-Hu Chen, Kun Zhang, Li-Ming Lu

**Affiliations:** ^1^Medical College of Acu-Moxi and Rehabilitation, Guangzhou University of Chinese Medicine, Guangzhou, China; ^2^Department of Acupuncture and Moxibustion, The Third Affiliated Hospital of Sun Yat-sen University, Guangzhou, China; ^3^Department of Prevention and Health, The Third Affiliated Hospital of Sun Yat-sen University, Guangzhou, China; ^4^Department of Acupuncture, The First Affiliated Hospital of Guangzhou University of Chinese Medicine, Guangzhou, China; ^5^South China Research Center for Acupuncture and Moxibustion, Medical College of Acu-Moxi and Rehabilitation, Guangzhou University of Chinese Medicine, Guangzhou, China

**Keywords:** study protocol, randomized controlled trial (MeSH), acupuncture, Parkinson's disease, Qihuang Needling therapy

## Abstract

**Background:**

Although significant progress has been made in the pharmacologic management of Parkinson's Disease (PD), effective management of movement disorders is still a hurdle for therapeutics targeting PD. Acupuncture is one therapeutic option that could potentially improve the motor function of PD and is widely used as adjuvant therapy. Among the various acupuncture approaches, *Qihuang Needling* (QHN) therapy has been found to improve motor-function control for patients with PD. However, evidence regarding its efficacy remains scarce. Therefore, to address this need, this study will determine the effects of QHN therapy on motor function in patients with PD and compare it to placebo effects.

**Methods:**

This trial is a multicenter, prospective randomized controlled clinical trial. We randomly allocated 144 participants to two groups of 72 patients. Patients in the treatment group were treated with QHN therapy. The control group had undergone insertion of acupuncture needles at sham acupoints not corresponded to acupuncture points. Participants in the verum treatment group and sham-acupuncture control group received 9 sessions over 6 weeks followed by 8 weeks of follow-up. The primary outcome was the change of motor function from baseline to weeks 6 and 14 measured by the PD Rating Scale-Part III Motor Examination (UPDRS-III). Secondary outcome measures included the change of PD daily quality of life-39 (PDQ-39) and Non-Motor Symptoms Scale for PD (NMSS) from baseline to weeks 6 and 14.

**Discussion:**

The results of this trial will generate data to improve our general understanding of the efficacy of QHN therapy on motor function in patients with PD and thoroughly compare these responses to the placebo effect.

**Trial Registration:**

The trial was registered at the Chinese Clinical Trials Registry (ChiCTR- 2000030871) on 16 March 2020.

## Background

Parkinson's disease (PD) is a progressive neurodegenerative condition in which patients present clinical motor symptoms, such as bradykinesia, rigidity, resting tremor of distal extremities, and postural instability (1). Additionally, patients with PD may develop a series of non-motor symptoms, such as depression, cognitive impairment, sleep disorder, and autonomic disturbance (2). Unfortunately, PD has become the second-most common neurodegenerative disorder that affects estimated 6 million people worldwide (3).

The core pathological changes of PD lie in intracellular inclusions containing aggregates of α-synuclein and striatal dopamine deficiency induced by the loss of neurons in the substantia nigra. With the discovery of the role of dopamine deficiency in PD, pharmacologic dopamine substitution treatment became the foundation of current drug therapies for PD (4). In addition to oral medications, surgical intervention, such as deep brain stimulation, has been beneficial to patients with troublesome motor fluctuations and dyskinesia due to the advanced stages of PD. However, there remain challenges in developing effective treatment options for PD with these advancements. Due to none of these treatments effectively modifies disease progression and/or delay disability, patients with PD can often suffer from progressive degeneration of motor function. As the benefits of medications wane over time with disease progression, effective management of tremor, gait, balance, posture, and dexterity are major challenges for therapeutics designed for PD movement disorders (5). Beyond these unmanaged motor symptoms, many non-motor symptoms add considerably to the overall burden of the disease (6).

Acupuncture has existed for over 4,000 years as one of the main treatments of traditional Chinese medicine. As an adjuvant therapy, acupuncture is widely used in the treatment of PD, particularly in East Asia (7). Numerous clinical trials have shown that acupuncture could improve motor symptoms and the quality of sleep, reduce the dose and frequency of anti-PD drugs, and alleviate their side effects (8–11). However, none of these studies could verify whether the benefits are due to the efficacy of the treatment or as a result of the placebo effect (12, 13).

The different operating modes of acupuncture can be divided into manual acupuncture (MA) and electroacupuncture (EA). Based on the preliminary clinical observation, *Qihuang Needling* (QHN, a kind of MA technique based on traditional meridian theory) therapy has shown promising benefits targeted against better control for PD. However, the evidence for its efficacy remains scarce. Therefore, to address this gap, we aim to investigate the efficacy of QHN therapy when compared with sham acupuncture in patients with PD.

### Trial Design

For this study, we designed a multicenter, assessor and statistician blinded, randomized controlled trial (RCT) to compare QHN therapy with sham acupuncture in patients with PD. Trials were conducted in three medical centers: the First Affiliated Hospital of Guangzhou University of Traditional Chinese Medicine, the Third Affiliated Hospital of Sun Yet-Sen University, and the Guangdong 999 Brain Hospital.

**Figure 1:**
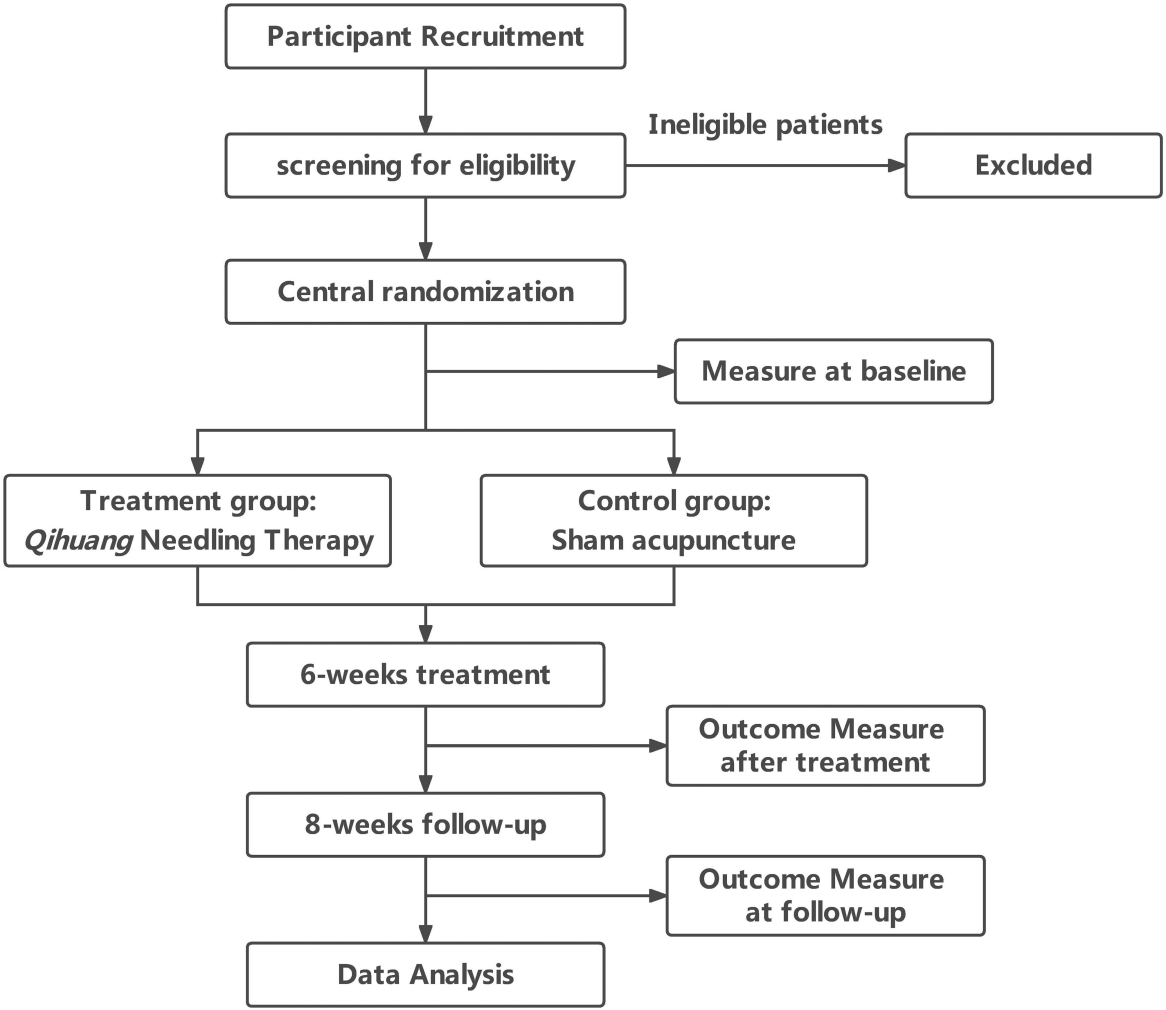
Trial flow chart.

A total of 144 patients with PD were recruited and randomly assigned to either the QHN therapy group (treatment group) or sham-acupuncture group (control group) at a 1:1 ratio (Figure 1). Our study was conducted according to the Declaration of Helsinki, and the Standard Protocol Items: Recommendations for Interventional Trials (SPIRIT) checklist is given in Supplementary File 1.

### Inclusion Criteria

The inclusion criteria in this study are as follows: (1) patients with PD, (2) patients of the age of 40–80 years old, (3) patients with a duration of PD of more than 1 year; (4) modified Hoehn-Yahr (HY) grades from 1 to 4; (5) patients who needed to take a stable dose of anti-PD drugs for more than 2 months, if treated with anti-PD drugs, or patients who had not received anti-PD medication for more than 2 months; (6) had clear consciousness and stable vital signs; and (7) patients who fully understood the study protocol and signed the consent form.

### Exclusion Criteria

Patients with any of the following criteria were excluded: (1) patients with severe hepatorenal disease, blood disease, tumor, endocrine disease, and/or infection. (2) Those who participated in other clinical trials during the same period; (3) patients with schizophrenia or other mental disorders that affected the compliance of patient; (4) those who were deaf or had communication difficulties caused by dementia; and (5) those who had a history of alcohol or drug abuse.

### Recruitment

Participants were recruited by advertisements on bulletin boards located at the Department of acupuncture and the Department of Neurology at the First Affiliated Hospital of Guangzhou University of Chinese Medicine, Guangdong 999 Brain Hospital, and the Third Affiliated Hospital of Sun yet-sun University. An independent assessor working in these departments was responsible for screening and registering the participants that met this study's inclusion criteria. The details of the participants were conserved by the Data Monitoring Committee (DMC) to ensure patient confidentiality.

### Randomization and Allocation Concealment

Central randomization was performed by the College of Acupuncture and Rehabilitation, Guangzhou University of Traditional Chinese Medicine. The random assignment operation was programmed and executed by using the SAS9.2 software. An independent researcher received the random numbers and group assignment after inputting the patients' information through an online application.

### Ethical Requirements and Registration

This study protocol was approved by the Ethics Committee of the Guangzhou University of TCM (Guangzhou, China) in July 2019 and was designated with permission number K2019-007. The trial was registered in the Chinese Clinical Trial Registry with approval number ChiCTR2000030871.

### Blinding

All evaluations were blinded. The person in charge of the efficacy evaluation was hired separately and was not aware of the grouping situation of the patients. The person in charge of the data analysis was also hired separately and did not participate in the subjects' specific clinical implementation work and/or design scheme.

### Interventions

To ensure the participants' safety and compliance and fulfill ethics necessities, we followed the recommendation of the Chinese Guidelines for the management of patients with PD (14). All participants in two groups received Madopar, whose dosage was recorded in detail. The original drug solutions and dosage were changed if the patients had already taken the anti-Parkinson's medicine before recruitment. In some cases, if the medications needed to be altered, the details were recorded carefully, such as the drug's name, administration time, and dosage. The whole treatment was carried out by licensed acupuncturists who had more than 5 years of clinical experience.

### Treatment Group

**Figure 2:**
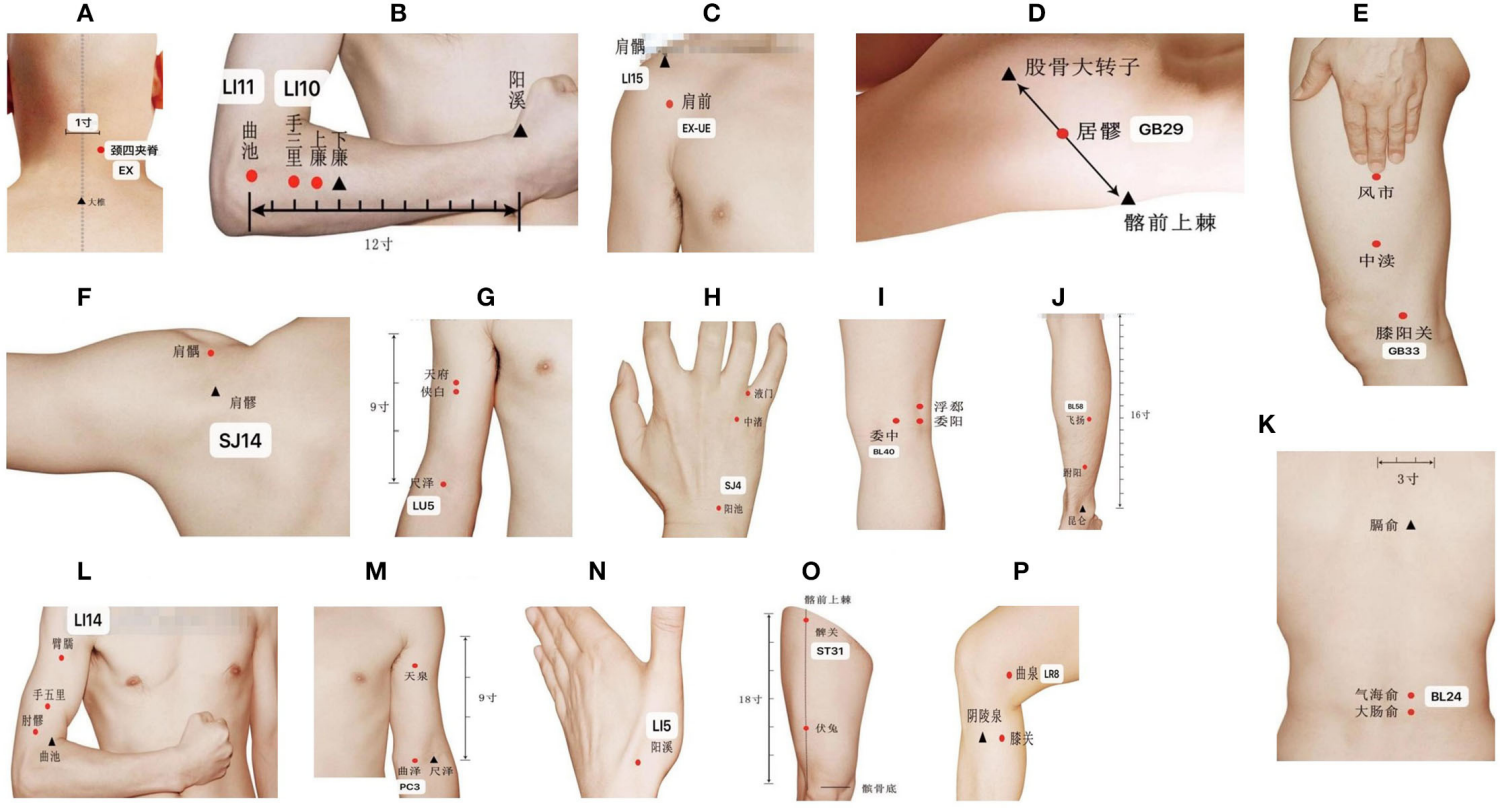
Location of acupoints. **(A)** EX-B2; **(B)** LI10; **(C)** EX-UE & LI15; **(D)** GB29; **(E)** GB33; **(F)** SJ14; **(G)** LU5; **(H)** SJ4; **(I)** BL40; **(J)** BL58; **(K)** BL24; **(L)** LI14; **(M)** PC3; **(N)** LI5; **(O)** ST31; **(P)** LR8.

**Figure 3:**
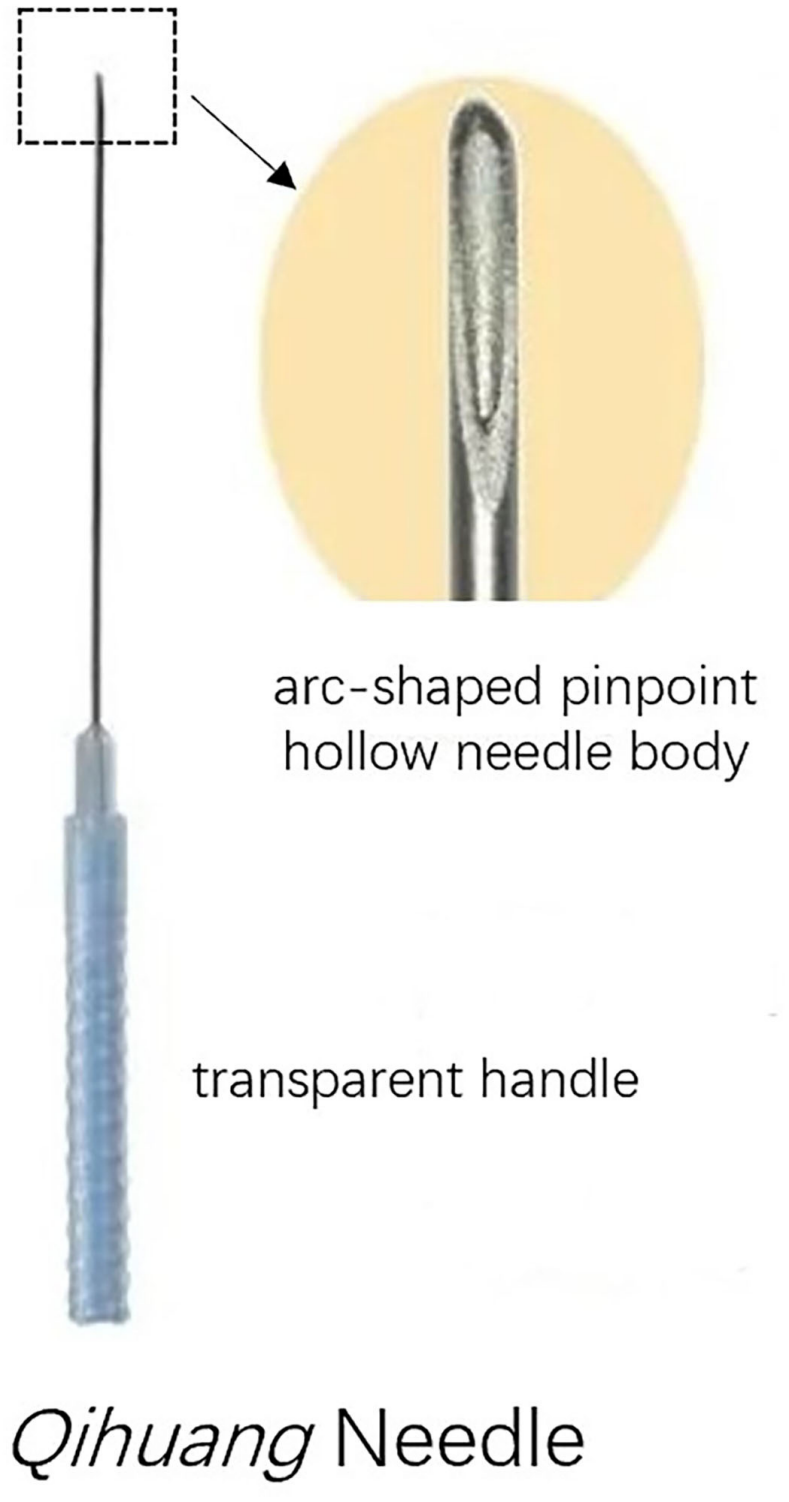
*Qihuang* Needle.

The design of the treatment group was based on the theory of Traditional Chinese Medicine. In addition to the standard routine care, the treatment group received QHN therapy. The location and needling methods for acupoints are demonstrated in Table 1, Figure 2, which were located in four limbs, the neck, and the back. A tailored sterile, stainless-steel needle (length: 50 mm; diameter: 0.5 mm; QH; Chongqing, Figure 3) was inserted into the described acupoints at a depth of 25–40 mm. After the patients felt the *Deqi* sensation, the needles were removed and re-applied at a 30° angle. All needles were withdrawn with clean cotton balls pressed to the skin to prevent bleeding. Participants received a total of nine sessions of treatments within 6 weeks. Treatment was given 2 times per week for the first 3 weeks and 1 time per week for the following 3 weeks.

**Table 1:** Location of acupoints in the treatment group.

**Session**	**Acupoints**	**Location**
1, 4, 7	EX-B2	0.5 *cun* lateral to the depression below the spinous process of the 4th cervical vertebra
	LI 10	on the dorsal-radial side of the forearm, 2 *cun* inferior the transverse crease of the elbow, on the line joining LI5 and LI11
	EX-UE	on the midpoint of the line between the top of anterior axillary folds and LI15 acupoint
	GB 29	at the lateral gluteal, the midpoint of the line between anterior superior iliac spine and the most convex point of the greater trochanter
	GB 33	on the lateral side of the knee, the depression above the external epicondyle of the femur
2, 5, 8	SJ14	in the depression posteroinferior to the acromion when arm is abducted
	LU5	on the transverse cubital crease, the radial side of the tendon of the biceps brachii
	SJ4	in the dorsal of the transverse crease of the wrist, the depression of the ulnar border of the total extensor tendon
	BL24	1.5 *cun* lateral to the depression below the spinous process of the 3th lumbar vertebra
	BL40	the midpoint of the transverse crease of the popliteal fossa
	BL58	on the lateral of the calf, 7 *cun* above the BL60 acupoint
3, 6, 9	LI14	7 *cun* above the transverse crease of the elbow, on the line joining LI11 and LI15
	PC3	on the transverse cubital crease, the depression of the ulnar border of the tendon of biceps brachii
	LI5	at the dorsal transverse crease of the wrist, at the depression between the tendons of the short extensor and long extensor of the thumb when the thumb is upward
	ST31	In anterior of the thigh, flush with the transverse crease of the hips, on the line joining the anterior superior iliac spine and the lateral side on the bottom of the patella
	LR8	on the medial side of transverse crease of the knee, the posterior edge of the medial condyle of the femur when bending the knee

^*^Additional points could be chosen according to syndrome differentiation.

*(1)constipation: ST25: (2) insomnia: BL14*.

### Control Group

The control group of this study was provided sham-acupuncture therapy. Sham acupoints are defined as 20-mm lateral of the verum acupoints. To do so, a needle was inserted into the sham acupoints at a depth of 3 mm without any subsequent manipulation. At these points, the patient did not feel the *Deqi* sensation. Similar to the treatment group, sham treatment was given 2 times per week for the first 3 weeks and 1 time per week for the following 3 weeks.

### Outcome Measurements

All outcome measurements were taken at baseline (before treatment), 6 weeks after treatment, and 14 weeks after treatment.

### Primary Outcome Measurement

#### Parkinson's Disease Rating Scale-Part III Motor Examination (UPDRS-III)

Parkinson's Disease Rating Scale-Part III Motor Examination-III is an assessor-rated scale that is used to evaluate the motor function of patients with PD. It contains 18 items, and each item is scored “0–4” depending on the severity of the motor disability, in which “0” represents normal and “4” indicates severe impairment. Higher scores indicate severe motor function and disability. This scale effectively assesses the severity of PD and has demonstrated high efficiency, validity, and reliability (15).

### Secondary Outcome Measurement

#### Parkinson's Disease Daily Quality of Life-39 (PDQ-39

Parkinson's disease daily quality of life-39 is a questionnaire used to assess the health-related quality of life (HRQoL) of patients with PD (16). This questionnaire contains 39 questions and evaluates eight dimensions: mobility, activities of daily living, emotional wellbeing, stigmatization, social support, cognition, communication, and bodily discomfort. Each question has five choices, in which higher scores indicate higher incidence rates of the physiological or psychological status of the patients, suggesting a low HRQoL.

### Non-Motor Symptoms Scale for Parkinson's Disease (NMSS)

**Non-Motor Symptoms Scale for Parkinson's disease** is a 30-item scale for assessing the non-motor symptoms of PD. It is used to evaluate cardiovascular symptoms, sleep, cognition, memory, dysesthesia, gastrointestinal symptoms, and urinary symptoms. Each item is rated using a “1–3” and “1–4” scale, where the former represents the severity of the non-motor symptom, whereas the latter indicates its frequency. Higher scores indicate a more severe non-motor disorder. Validation of this tool has been shown by excellent correlations between the H-Y scale and UPDRS scores.

### Sample-Size Calculation

This trial used a clinical-superiority design to verify that QHN therapy's effect was superior to that of sham acupuncture. The primary outcome measure was the UPDRS-III score difference before and after the treatment. According to previous research (17), assuming the standard deviation (SD) to be 8.0 and the mean of the treatment effect of the two groups to be 4.36 and 0.25, respectively, the statistical power was 80%, and the significance level was 0.05. Using PASS software requires each group to contain no <61 patients. With an estimated 15% dropout rate, we planned to recruit 72 patients for each group, for a total of 144 patients.

### Statistical Analysis

We used mean and SD for normally distributed variables, or median (interquartile) for the variables not normally distributed, to summarize the participants' demographic, health conditions, and clinical outcomes at three different time points. Two statisticians, blinded to the group setting, analyzed the data independently *via* SPSS software (version 26.0). Missing data were imputed according to the last-observation-carried-forward (LOCF) principle. Furthermore, the data were analyzed by the intent-to-treat principle.

The normality of the variables was assessed using the normal probability plot. The continuous variables normally distributed were assessed by the student's t-test. Otherwise, the Mann-Whitney test or Wilcoxon test was applied. The Fisher's exact or the chi-square test was adopted for categorical data, and statistical significance was set at *p* < 0.05.

### Safety and Adverse Events (AEs)

Acupuncture is generally regarded as a safe therapy. Although AEs of acupuncture are rare, participants still may encounter hematoncus, dizziness, or fainting. If the above events occur, the acupuncture treatment will be stopped immediately, and the subject will be instructed to lie flat on the bed and drink warm water. AEs are caused by oral drugs that mainly include nausea, vomiting, dyspepsia, abdominal distension, orthostatic hypotension, hypohepatia, and renal function impairment. If these adverse reactions occur, participants will halt the use of medication, the respective alternative medicine, and symptomatic treatment as necessary. These AEs will be subcategorized by severity: mild, moderate, and severe AEs. For this study, mild AEs were defined as transient and tolerable AEs. Moderate AEs were defined as those that caused discomfort and potentially interfered with the subject's daily life. Severe AEs were defined as those that seriously affected the participants' physical health and even led to the risk of life. The details of AEs, such as time, duration, performance, measures to be taken, and the outcome, were recorded. The trial was stopped if there was an unacceptable risk of serious AEs in one or both treatment arms.

### Data Management and Monitoring

A case report form (CRF) was designed and utilized for data collection. The DMC recorded data information regarding subject demographics and assessment of patients. As necessary, the reason for patient dropout was documented in the CRF. At the end of the study, the investigator submitted all CRFs to the data management committee for review.

If more than 25% of the patients stopped treatment due to moderate or severe AEs, trial continuation would be required to be reassessed. The DMC was independently chaired by the Statistics Teaching and Research Office of Guangzhou University of Chinese Medicine and claimed to have no conflict of interest. The South China Research Center for Acupuncture and Moxibustion acted as an independent committee to monitor the progress and provide advice. The Ethics Committee of the First Affiliated Hospital of Guangzhou University of Chinese Medicine took part in endpoint adjudication.

For the duration of this study, the Project Management Group met every week to review trial conduct. Likewise, the Trial Steering Group met every month, and the independent Data Monitoring and Ethics Committee met every 6 months to review conduct throughout the trial period.

## Discussion

Previous investigations have investigated the efficacy of acupuncture for PD, with data supporting using these methods to benefit patients with PD (18, 19). Our findings from this study will further support our general understanding of therapeutic options and elucidate the benefits of QHN therapy in terms of improving motor symptoms, such as postural instability and functional mobility. If identified that it effectively alleviates motor symptoms and improves HRQoL, it could serve as a low-cost adjuvant therapy for patients with PD with motor symptoms.

This research will also evaluate the efficacy of QHN therapy for non-motor symptoms of PD, such as chronic constipation and insomnia. In addition, we aimed to explore alterations in regional brain activity before and after therapy through Functional brain imaging techniques.

*Qihuang Needling* therapy adopts a tailored disposable sterile needle characterized by its arc-shaped pinpoint, hollow-stiff needle body, and transparent handle. Compared with ordinary traditional manual acupuncture (TMA), the advantages of QHN therapy are that it provides a stronger *Deqi* sensation at the acupoint, reduces puncture pain, and has shorter operation times. Furthermore, it is regarded as safer than TMA because an acupuncturist could identify bleeding directly through a transparent handle to avoid damage to blood vessels. In support of these benefits, previous trials have shown that the efficacy of QHN therapy for musculoskeletal diseases is superior to TMA (20–23).

Several studies have demonstrated that acupuncture is widely used for Chinese patients with PD (7). Additionally, our previous clinical observation indicates that QHN therapy may have sustained effects, which may be superior to other acupuncture approaches. However, evidence for the therapeutic effects of acupuncture is limited. A review study summarizing 35 investigations in mainland China, Japan, Korea, Taiwan, and the United States of America demonstrated that most of these trials had small sample sizes, and some were individual case reports (10). Additionally, many studies are limited by methodological approaches, such as the lack of a placebo control group and/or deficiencies in their randomized design. To further elucidate QHM efficacy and address these previous research limitations, this research uses a multicenter, randomized, placebo-controlled trial with 400+ subjects to provide advantages in methodological design.

### Limitations

Our study has several recognized limitations. Firstly, it is an open-label research investigation due to the inability of blind acupuncturists. Despite blinding assessments being performed, we did not control for patients' and acupuncturists' expectations of efficacy, which may alter the results of the blinding assessment and the effects of QHN therapy. Secondly, the longer-term follow-up has not been well assessed because PD is a chronic and progressive condition. Thirdly, limited by the expenditure, gait examination will not be performed in patients with PD. Therefore, future clinical trials may need to include taking gait examination as the primary outcome, and a comparison to standard care and a waiting list.

## Trial Status

This protocol is version 2.0. 2019-03-17. The participants will be recruited from 1 March 2022 to 1 June 2023.

## Ethics Statement

The studies involving human participants were reviewed and approved by the Ethics Committee of the Guangzhou University of Traditional Chinese Medicine (Guangzhou, China) in July 2019 (K2019-007). Written informed consent will be obtained from eligible participants before any assessment or intervention.

## Author Contributions

L-SY and Y-ML drafted the manuscript. D-FZ edited the manuscript. L-SY, D-FZ, and B-MZ performed the intervention. Y-ML and S-ZZ collected the data and helped to statistical analysis. Z-HC, KZ, and L-ML carried out the design of the study. All authors issued final approval for the version to be submitted.

## Funding

Funding for this work comes from the Administration Bureau of Guangdong Academy of Traditional Chinese Medicine (no. 20203014).

## Conflict of Interest

The authors declare that the research was conducted in the absence of any commercial or financial relationships that could be construed as a potential conflict of interest.

## Publisher's Note

All claims expressed in this article are solely those of the authors and do not necessarily represent those of their affiliated organizations, or those of the publisher, the editors and the reviewers. Any product that may be evaluated in this article, or claim that may be made by its manufacturer, is not guaranteed or endorsed by the publisher.

## Abbreviations

PD: Parkinson's Disease
QHN: Qihuang Needling
RCT: Randomized controlled trial
AEs: Adverse events
CRF: Case report form
HY: Hoehn-Yahr
DMC: Data Monitoring Committee
UPDRS-III: Parkinson's Disease Rating Scale-Part III
PDQ-39: Parkinson disease daily quality of life-39
NMSS: Non-Motor Symptoms Scale for Parkinson's disease
SD: standard deviation
AEs: adverse events.
