# Supplementary material for: Effects of Qihuang Needling on Motor Function for Patients With Parkinson's Disease: Study Protocol for a Multicenter, Randomized Controlled Trial
Source: Front Neurol. 2022 Jun 2;13:902170. doi: 10.3389/fneur.2022.902170 (PMC9201048; doi:10.3389/fneur.2022.902170)
Supplement: Supplementary file 1 [file Table_1.docx]

**Supplementary Content**

| Session | acupoints | Location |
| --- | --- | --- |
| 1, 4, 7 | EX-B2 | 0.5 *cun* lateral to the depression below the spinous process of the 4^th^ cervical vertebra |
|  | LI 10 | on the dorsal-radial side of the forearm, 2 *cun* inferior the transverse crease of the elbow, on the line joining LI5 and LI11 |
|  | EX-UE | on the midpoint of the line between the top of anterior axillary folds and LI15 acupoint |
|  | GB 29 | at the lateral gluteal, the midpoint of the line between anterior superior iliac spine and the most convex point of the greater trochanter |
|  | GB 33 | on the lateral side of the knee, the depression above the external epicondyle of the femur |
| 2, 5, 8 | SJ14 | in the depression posteroinferior to the acromion when arm is abducted |
|  | LU5 | on the transverse cubital crease, the radial side of the tendon of the biceps brachii |
|  | SJ4 | in the dorsal of the transverse crease of the wrist, the depression of the ulnar border of the total extensor tendon |
|  | BL24 | 1.5 *cun* lateral to the depression below the spinous process of the 3^th^ lumbar vertebra |
|  | BL40 | the midpoint of the transverse crease of the popliteal fossa |
|  | BL58 | on the lateral of the calf, 7 *cun* above the BL60 acupoint |
| 3, 6, 9 | LI14 | 7 *cun* above the transverse crease of the elbow, on the line joining LI11 and LI15 |
|  | PC3 | on the transverse cubital crease, the depression of the ulnar border of the tendon of biceps brachii |
|  | LI5 | at the dorsal transverse crease of the wrist, at the depression between the tendons of the short extensor and long extensor of the thumb when the thumb is upward |
|  | ST31 | In anterior of the thigh, flush with the transverse crease of the hips, on the line joining the anterior superior iliac spine and the lateral side on the bottom of the [patella](javascript:;) |
|  | LR8 | on the medial side of transverse crease of the knee, the posterior edge of the medial condyle of the femur when bending the knee |

Table 1: Location of acupoints chose in trial group

* Additional points could be chosen according to syndrome differentiation：


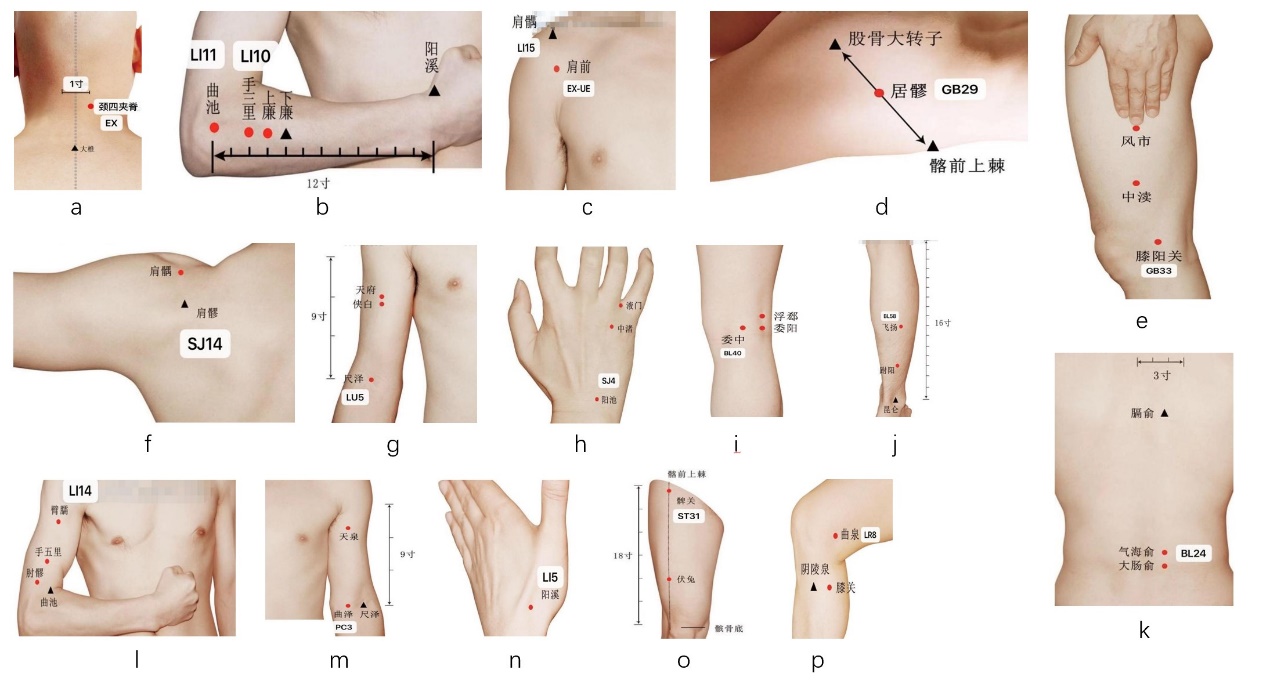
（1）constipation：ST25；（2）insomnia: BL14
